# Employing artificial intelligence to predict δ¹⁸O and δ²H isotope ratios in precipitation in Iraq under changing climate patterns

**DOI:** 10.1038/s41598-026-35047-x

**Published:** 2026-01-08

**Authors:** Ali Al Maliki, Ali Al-Naji, Ahmed Kadhim Al Lami, Haitham Abdulmohsin Afan, Maryam Bayatvarkeshi, Nadhir Al-Ansari

**Affiliations:** 1 Scientific Research Commission, Baghdad, Iraq; 2 College of Engineering, Al-Ayen University, Thi-Qar, Iraq; 3 Electrical Engineering Technical College, Middle Technical University, Baghdad, Iraq; 4 Department of Physics, College of Science, Al-Nahrain University Jadriya, Baghdad, 10072 Iraq; 5 Upper Euphrates Center for Sustainable Development Research, University of Anbar, Ramadi, 31001 Iraq; 6 Department of Geography and Environmental Management, University of Waterloo, Waterloo, ON Canada; 7 Civil, Environmental and Natural Resources Engineering, Lulea University of Technology, Lulea, 97187 Sweden

**Keywords:** Deuterium, Oxygen-18, Precipitation, Environmental isotope, Machine learning, Iraq, Climate sciences, Environmental sciences, Hydrology

## Abstract

Understanding precipitation dynamics in arid regions such as Iraq is of paramount importance in hydrological and climatological studies, as it is a key approach to water resources management and climate change adaptation. This study aims to develop a mathematical predictive model for rainfall isotopic values using machine learning techniques. Stable isotope data for oxygen (δ¹⁸O) and deuterium (δ²H) in precipitation were collected from 32 meteorological stations distributed across Iraq over a 14-year period (2010–2024). The dataset also included meteorological parameters for these stations, including precipitation amount, air temperature, relative humidity, and calculated station elevation. Several machine learning algorithms (i.e., SVM, GBR, ANN, CatBoost, XGBoost, and RF) were employed to compare predicted isotopic values with actual readings, accounting for rainfall characteristics and patterns. The results demonstrated that the RF model achieved superior predictive performance, with a calibration coefficient (R²) of 0.89 in the testing set, indicating strong predictive capability. This model also recorded the lowest mean absolute error (MAE) of 1.39 and the lowest root mean square error (RMSE) of 3.5 compared to the other algorithms, reflecting improved predictive accuracy. These findings confirm the effectiveness of integrating machine learning, particularly the RF approach, in enhancing the modeling of isotopic signature predictions in environmental studies. Furthermore, they highlight the potential of AI-based models as powerful tools for reconstructing historical isotopic datasets, supporting climate variability assessment and sustainable water resources management in arid and semi-arid regions.

## Introduction

Precipitation is the most important component of the water cycle, which plays as a principal role in hydrological and climatological systems^1^. Climate changes affect the hydrological cycle and the flow systems of waterways, which in turn change the volume of water available locally, regionally, and globally at various levels^2^.

Using precise proxies like stable isotopes (δ^18^O and δ^2^H) is a key component in assessing, controlling, and protecting water resources^3^. The main variables that affect isotope ratios in precipitation are climate and location^4^. The use of stable isotopes to solve biogeochemical problems in ecosystem analysis is increasing rapidly because stable isotopes data can contribute both source-sink (tracer) and process information^5^. Stable environmental isotopes use as a valuable tool for studying the origins of water bodies, allowing for a good understanding of their capacity and more efficient utilization^6^. These stable isotopes of hydrogen and oxygen (δ2H and δ18O) provide a distinct signature for each water source. This enables us to evaluate their sources, assess contamination risks, and investigate the movement and fate of pollutants. Additionally, these isotopes can also provide insights into the paleoclimatic conditions during the time of water recharge. A comprehensive study was conducted across different regions of Iraq to establish an isotopic database of Deuterium (δ²H) and Oxygen-18 (δ¹⁸O) in groundwater and to examine its relationship with the isotopic signature of surface water^7^.

The factors driving variability in rainfall stable water isotopes (specifically δ^18^O and deuterium excess, d = δ^2^H − 8 δ^18^O) were studied in a 13-year data set of daily rainfall samples from coastal southwestern Western Australia (SWWA). Backwards dispersion modeling, automatic synoptic type classification, and a statistical model were used to establish reasons for variability on a daily scale. The predictions from the model were accumulated to longer temporal scales to find out the cause of variability on multiple timescales^8^.

Artificial intelligence (AI) offers a powerful toolkit for unraveling these intricate relationships and gaining deeper insights. Forty-two precipitation sampling stations were chosen across the Islamic Republic of Iran to assess the fractional importance of these climatic and geographic factors influencing stable isotopes. Additionally, deep learning models were employed to simulate the stable isotope content, with missing data initially addressed using the predictive mean matching (PMM) method. Deep neural network (DNN) models were utilized to predict stable isotope values in precipitation, AND validation using evaluation metrics demonstrated that the model based on DNN exhibited higher accuracy^9^. This study underscores the efficacy of ML techniques in both simulating and forecasting stable isotope contents with high precision. Several models, including Artificial Neural Networks (ANNs), Stepwise Regression, and Ensemble Machine Learning approaches, were applied to simulate stable isotope signatures in precipitation. Among the studied Machine Learning Models, XGboost showed the most accurate simulation with higher R^2^ (0.84 and 0.86) and lower RMSE (1.97 and 12.54), NSE (0.83 and 0.85), AIC (517.44 and 965.57), and BIC values (531.42 and 979.55) for ^18^O and Deuterium compared to other models, respectively^10^.

The link between environmental isotopes and changing rainfall patterns is a complex and fascinating topic, holding immense potential for understanding climate change and water resource management.

Quantile regression forest for Estimating Evapotranspiration Rates has been used in Iraq, using an RBF-NN artificial intelligence model to estimate evapotranspiration rates across different regions of Iraq based on climatic parameters and vegetation cover^11,12^.

The model provided robust estimations compared to traditional methods. This technique was aiding in efficient water resource management. Developing a framework using machine learning to calculate isotope time series at a monthly resolution using available climate and location data. As a result, this can improve precipitation isotope model predictions, which can serve as resources for probing historic patterns in the isotopic composition of precipitation with a high level of meteorological accuracy^13^. A study suggested using data-driven methods, multilayer perception when lacking appropriate laboratory isotope analysis or facing high laboratory analysis costs. The determination coefficient (R^2^), mean absolute error (MAE), and root mean square error (RMSE) were used to evaluate the performance of the models. In addition, visualization techniques (e.g., Taylor diagram and heat maps) were prepared to assess the similarities between the measured and estimated δD and δ^18^O values^14^.

A previous study compared the performance of three artificial neural network (ANN) models—Radial Basis Function (RBF), Multilayer Perceptron (MLP), and Group Method of Data Handling (GMDH)—with the conventional Penman–Monteith (PM) method for estimating monthly reference evapotranspiration (ET₀) in Basrah City, southern Iraq^15^.

Several machine learning (ML) techniques were implemented, such as shallow neural network (SNN), deep neural network (DNN), decision tree (DT), random forest (RF), and extreme gradient boosting (XGBoost). XGBoost showed the highest accuracy across the majority of studied stations, with a R2 = 0.91, VNS = 0.90, AIC = 405, BIC = 410, and RMSE = 0.76. Additionally, DNN exhibited superior accuracy in specific cases, achieving a R2 = 0.87, VNS = 0.87, AIC = 445, BIC = 460, and RMSE = 1.10^16^.

To enhance predictive capabilities, a Support Vector Machine (SVM) model was adapted to estimate δ¹⁸O values using multiple hydrochemical indicators, achieving strong performance (R² = 0.92, MSE = 2.89). The study links directly to national water security goals and supports the global Sustainable Development Goal 6 (SDG 6) for clean water and sanitation^17^.

An efficient ML model based on an ensemble Deep random vector functional link (EDRVFL) optimized by a robust optimization method was developed to forecast daily AQI in three cities (Chengdu, Wuhan, and Taiyuan) in China^18^.

A study proposed an external attention-based ensemble learning method (EA-ensemble) that combines five sub-models, namely, XGBoost, RF, CNN, GRU, and MLP, for ET_O_ prediction^19^. A study was focused on providing an optimal solution for monitoring and accurate estimation of river streamflow using EML and ML techniques. The optimal weighted ensemble models were developed through the use of some influential algorithms^20^.

Despite the availability of various predictive techniques, few studies have evaluated their reliability under diverse and extreme climatic conditions. This study addresses this gap by applying and validating the methodology in the Iraq region, which is characterized by complicated climatic variability.

Our study showed the importance of implementing a particular study to integrate data obtained from the analysis of numerous rainwater samples collected over a long period, as well as data from previously published literature. Additionally, the study showed and assessed the impact of ambient temperature, elevation, and relative humidity on the measured values of stable isotopes of hydrogen and oxygen (δ^2^H and δ^18^O). The current methodology outlined holds a promising technique for application in regions worldwide characterized by diverse and severe climatic conditions. This study aims to develop a mathematical model to predict isotopic values using artificial intelligence techniques.

## Study area: location and climate

**Fig. 1:**
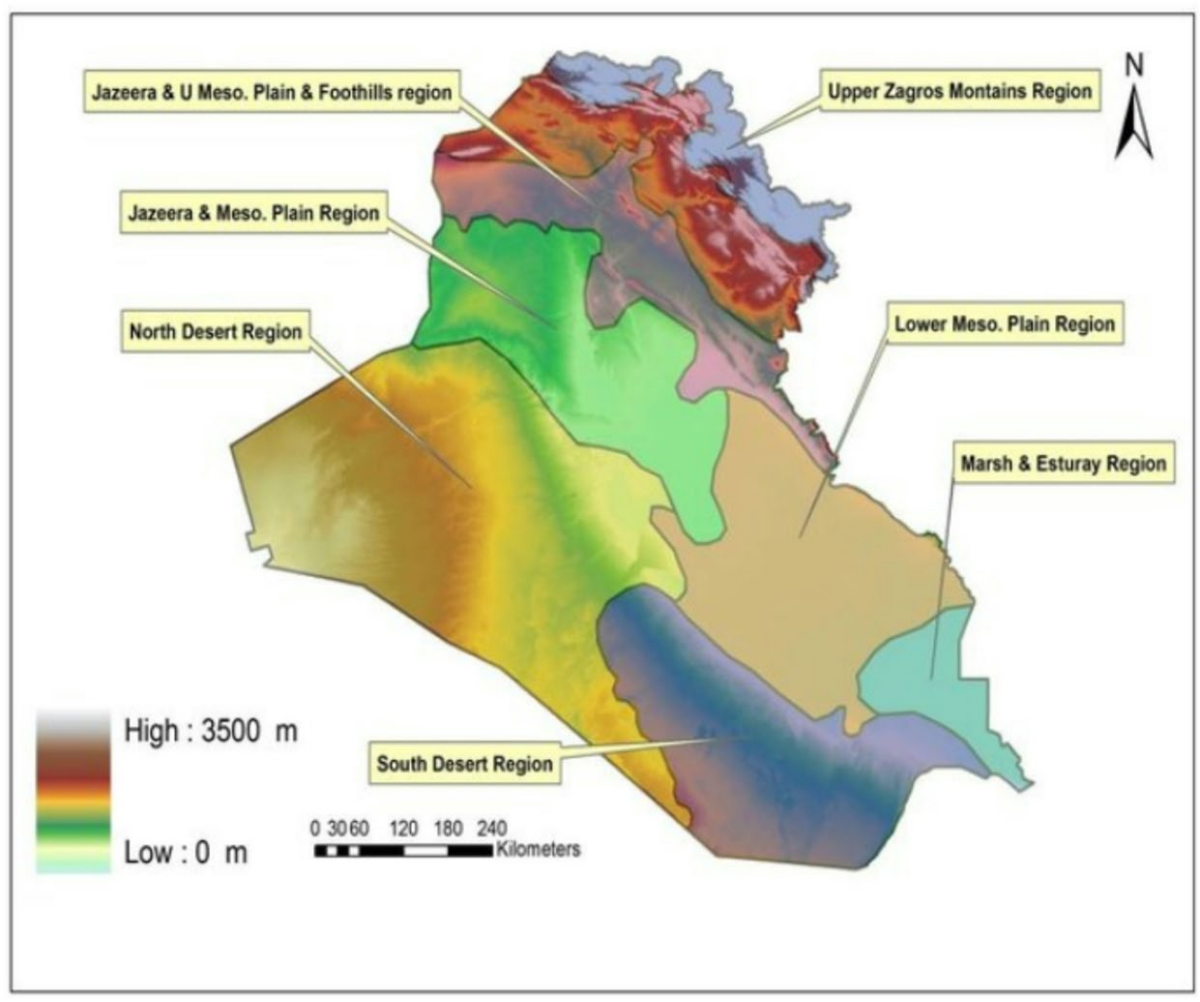
Major topographic regions of Iraq according to^24^.

Iraq is situated in Southwest Asia, where geographically located in the semi-arid region between latitudes (29.5 ^о^ −37.5 ^о^ N) north the equator, and between longitudes (38.45 ^о^- 48.45 ^о^ E) east of Greenwich line^21^. Figure 1 explains the six regions that make up the topography of the nation: Upper Zagros mountain region, near the borders with Iran and Turkey; Jazirah zone, north of Iraq between the Tigris and Euphrates rivers, which includes two regions, Upper Meso. plain and foothills region and Meso. plain region; Lower Meso. plain region, which represented the alluvial plains in the center and southeast, the North desert region, with the South desert region, which is located west of the Euphrates River, and the Marsh Estuary region^22^.

Due to its subtropical continental climate, which is arid to semi-arid, with hot, dry summers and nearly cold winters^23^, Iraq was chosen as a unique study area, where more than 70% of the country consists of arid and semi-arid regions^24^. Additionally, the climate varies in different part of Iraq, according to Bailey classification of humidity index, the climate of Iraq can be classified into three categories: semi humid zone in the far north (above 360 N), semidry zone (330–360 N), and dry zone in the middle and south of Iraq (under 330 N)^25^. The diversity of Iraq’s climate is attributed to several factors, the most prominent of which is Iraq’s astronomical location between latitudes (29–37) north^26^.

## Methodology

### Data preparation

**Fig. 2:**
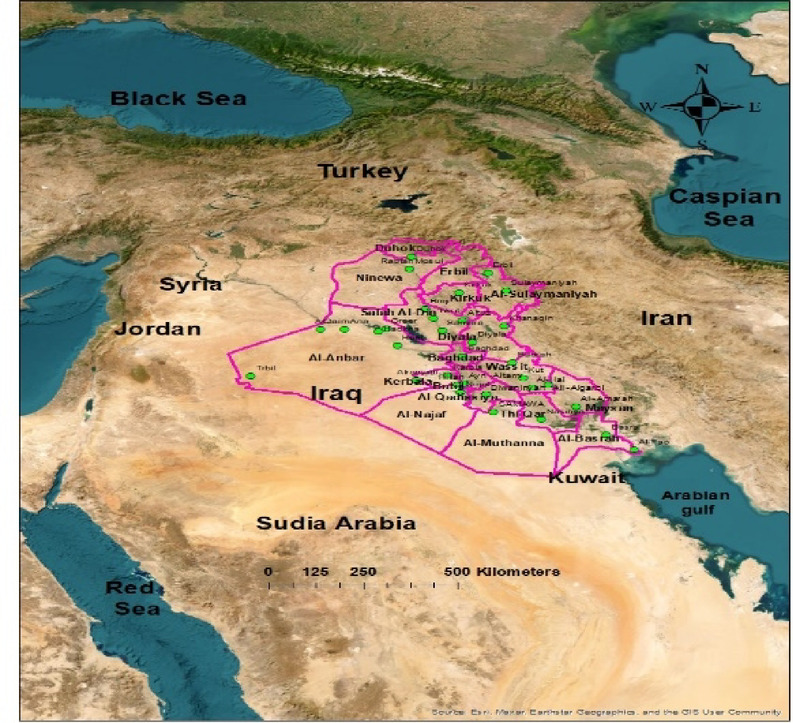
Geographic location of sampling stations across Iraq.

Stable isotope data for oxygen (δ¹⁸O) and deuterium (δ²H) in rainfall were collected from 34 stations distributed across Iraq over a 14-year period (2010–2024), as shown in Table 1. An isotopic database was obtained from the Water Isotope System for Data Analysis, Visualization, and Electronic Retrieval (WISER). This Website is the common access point to the International Atomic Energy Agency’s (IAEA) scientific, technical, and regulatory information resources^28,29^. The rain samples were collected during the rainy season (November to April) with the support of the Iraqi Meteorological Organization and Seismology, under the guidance of authorities from meteorological stations across various governorates in Iraq to accomplish this work. The spatial distribution of the weather stations, reflecting the different topography of Iraq, is illustrated in Fig. 2. These samples, as shown in Table 1, give the mean isotope values. The negative values for isotopes refer to water that is isotopically lighter than the standard, typically indicating depletion in the heavier isotopes (δ^18^O and δ^2^H) due to processes such as precipitation under colder climatic conditions or the removal of heavier isotopes through condensation or partial evaporation. Conversely, the positive values indicate samples that are isotopically heavier than the standard, often reflecting warmer conditions, increased evaporation, or interaction with surface waters in arid environments.

**Table 1:** Geographic location of sampling stations across Iraq and isotope weighted mean data for the period (2010–2024).

Station no.	Station name	Long (deg)	Lat (deg)	Altitude (m)	Sampling period	Sample no.	δ^18^O per mil, ‰	δ^2^H per mil, ‰
1	Sulaymaniyah	45.27	35.32	843	12/2011-2/2019	6	− 5.99	−32.74
2	Erbil	44.00	36.09	420	12/2011-1/2024	4	−6.81	−39.06
3	Duhok	43.05	36.84	565	12/2023	1	−0.54	10.16
4	Kirkuk	44.24	35.28	331	1/2012-3/2012	2	−5.79	−32.64
5	Mosul	43.09	36.19	223	12/2011-2/2024	12	−4.22	−17.16
6	Rabiah	42.06	36.47	382	11/2011-3/2024	7	−4.41	−20.98
7	Altuz	44.39	34.53	220	12/2023-2/2024	2	−2.54	−9.23
8	Baiji	43.32	34.54	115.5	12/2011-1/2012	2	−6.39	−34.12
9	Tikrit	43.42	34.34	107	12/2011-11/2023	4	−4.66	−20.75
10	Samara	43.54	34.11	75	1/2012-2/2018	5	−5.54	−29.06
11	Trbil	33.43	38.44	822	12/2011-3/2012	3	−6.05	−30.76
12	AlQaim	41.01	34.23	177.5	11/2023	1	−4.21	−15.69
13	Ana	41.57	34.22	174.5	11/2023	1	−1.31	−4.77
14	Haditha	42.21	34.08	108	12/2011-11/2023	4	−3.72	−13.86
15	Alramadi	43.19	33.27	48	12/2011-2/2024	7	−2.84	−9.06
16	Heet	43.45	33.38	58	2/2013-11/2023	3	−4.02	−11.83
17	Greer	42.566	34.237	100	12/2011-3/2012	3	−4.2	−18
18	Khanaqin	45.23	34.21	202	11/2011-2/2024	5	−3.6	−14.73
19	Badrah	45.57	33.06	64	11/2023-1/2024	2	−1.54	−3.72
20	Najaf	44.19	31.57	53	3/2013-11/2013	2	−4.17	−18.81
21	Diyala	44.6243	33.7633	48.78	12/2011-3/2012	3	−5.42	−28
22	Baghdad	44.24	33.18	31.7	1/2011-3/2024	38	−2.42	−4.31
23	Hillah	44.27	32.27	27	12/2023	1	0.67	11.32
24	Karbla	44.0335	32.34	29	12/2011-3/2012	3	−3.23	−11.1
25	Ayn -Altamr	44.43	32.33	27.9	10/2023-11/2023	2	−1.28	3.71
26	Al-Hai	46.02	32.08	17	11/2011-3/2012	4	0.97	2.83
27	Kut	45.49	32.3	19	12/2011-3/2012	3	−2.9	−11.8
28	Diwaninyah	44.57	31.57	20	12/2011-2/2024	5	−2.51	−3.99
29	Ali -Algarbi	46.43	32.28	14	11/2023-12/2023	2	−0.96	8.59
30	SAMAWA	45.16	31.16	11.4	1/2011-12/2013	3	−1.77	−1.56
31	Al -Amarah	47.1	31.5	9.5	12/2011-11/2023	4	−2.91	−11.07
32	Basra	47.47	30.31	2	11/2011-1/2016	8	−2.62	−8.18
33	Nasiriya	46.14	31.01	5	12/2011-3/2012	2	−2.95	−12
34	Al-Fao	48.4709	48.4709	2	1/2012-2/2012	2	−0.31	8.32

### Dataset and input features

The dataset comprised 279 samples, each with the following input features:

- Rain amount (precipitation amount).
- Temperature (Temp avg).
- Relative humidity (RH%).
- Elevation (m).

These variables were selected due to their known influence on isotopic fractionation in rainfall. A correlation test between all variables, as shown in Table 2 illustrates the relationship between these variables. The highest correlation between the inputs and the output is observed for elevation, followed by air temperature. The dataset was split into a training set with about 80% samples, and a test set with 20% samples. Moreover, six distinct machine learning algorithms were applied and compared in this study.

**Table 2:** Performance metrics of machine learning models.

ML Model	*R*² Score	MSE	MAE	RMSE	MAPE	EVS
SVM	0.17	140.94	6.54	11.87	156.78	0.18
GBR	0.75	34.68	3.30	5.89	63.92	0.76
ANN	0.79	32.25	3.04	5.68	60.63	0.79
CATBoost	0.81	26.78	2.90	5.17	55.51	0.81
XGBoost	0.87	17.10	2.07	4.14	37.90	0.87
Random Forest	0.90	12.93	1.39	3.60	18.75	0.90

ML Model : R² Score, Mean Squared Error (MSE), Mean Absolute Error (MAE), Root Mean Squared Error (RMSE), Mean Absolute Percentage Error (MAPE) %, Explained Variance Score (EVS)

Support Vector Machine (SVM): A powerful and versatile machine learning model capable of performing linear or non-linear classification, regression, and even outlier detection. SVMs work by finding the hyperplane that best separates different classes in the feature space.

Gradient boosting regressor (GBR): An ensemble learning method that builds a strong predictive model from a combination of weaker models, typically decision trees. It iteratively trains new models to correct the errors of previous models.

Artificial neural network (ANN): A computational model inspired by the structure and function of biological neural networks. ANNs consist of interconnected nodes (neurons) organized in layers, capable of learning complex patterns and relationships in data.

CatBoost: A gradient boosting library developed by Yandex. It is known for its ability to handle categorical features effectively and its robustness against overfitting.

XGBoost: An optimized distributed gradient boosting library designed to be highly efficient, flexible, and portable. It implements machine learning algorithms under the Gradient Boosting framework.

Random forest (RF): An ensemble learning method that operates by constructing a multitude of decision trees during training and outputting the mean prediction of the individual trees. It is highly effective for both classification and regression tasks and is known for its robustness and ability to handle high-dimensional data.

Minimal preprocessing was applied. The features were kept in their original units without scaling for clear interpretation. The 10% adjustment was used solely for data augmentation, where feature values were randomly shifted upward or downward by 10% to simulate natural noise and make the model more robust. The only exception was the ‘Elevation (m)’ feature, which was not changed during the augmentation, to ensure the geographical location of the data was preserved.

## Result and discussion

The modelling results have been summarized in Table 2 for each model type and compared across various performance indicators. This study found that the Random Forest (RF) model achieved exceptional results, outperforming other algorithms across all key metrics Table 2.

The hyper-parameter tuning for all machine learning models was conducted within a multi-output prediction structure, using 100 estimators and a random state of 42, which yielded the optimal performance metrics. The result indicates that the RF model achieved the highest predictive accuracy, with an R² value of 0.8983, explaining approximately 90% of the variability in isotopic composition. It also recorded the lowest error value (MAE: 1.39, RMSE: 3.60), signifying minimal deviation from actual isotopic values. Both XGBoost and CatBoost also performed well, but were outperformed by the RF model. In contrast, the SVM exhibited the weakest predictive capability, characterized by higher errors (MAE: 6.54) and a low R² (0.18), indicating limited ability to explain isotopic variability.

**Fig. 3:**
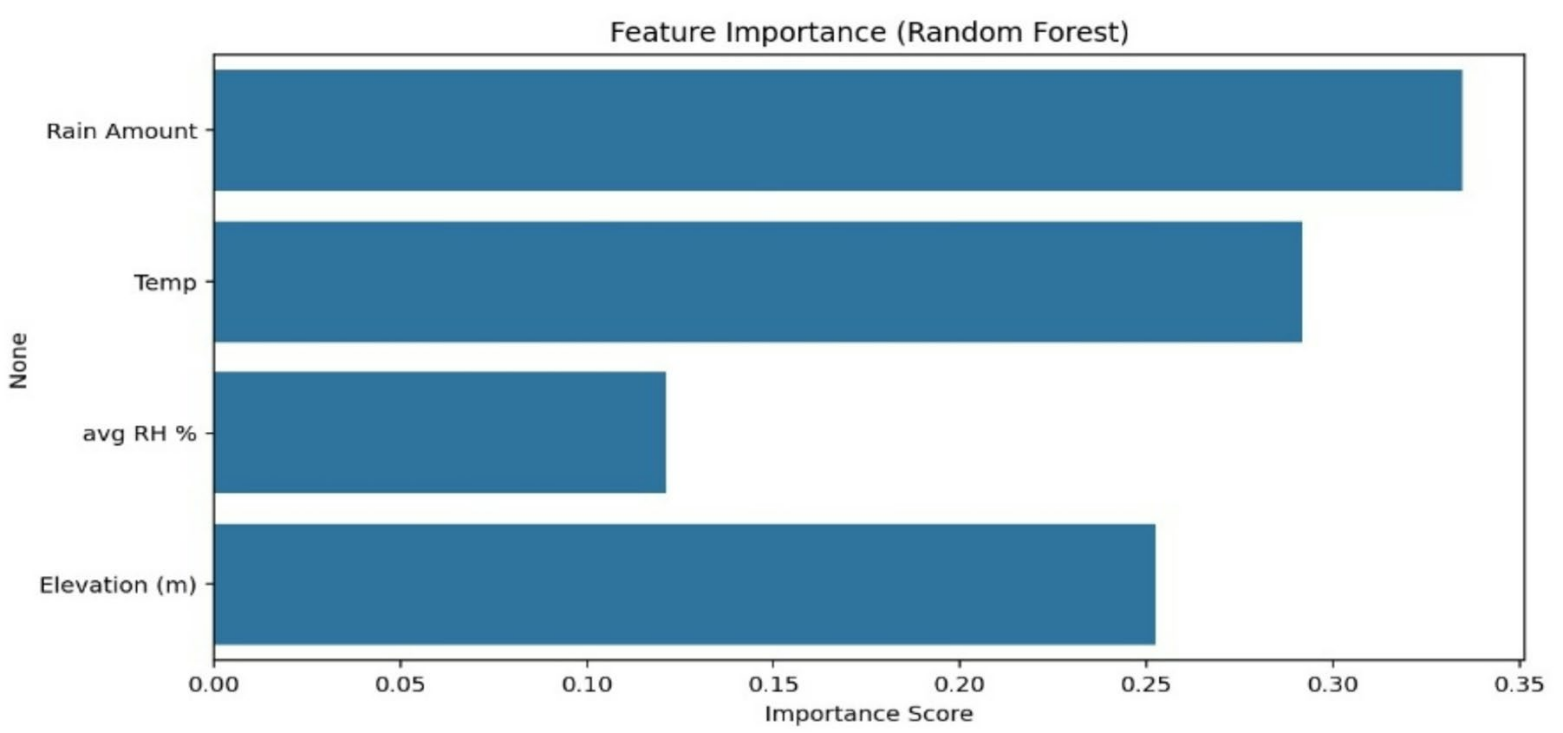
Feature importance (random forest).

The Random Forest model identified Rain Amount and Temperature as the most important features for predicting isotopes, followed by Relative Humidity and Elevation (see Fig. 3). Rain Amount (highest Impact) –Temperature- Elevation - Relative Humidity (lowest impact). This aligns with known hydroclimatic processes, where rainfall amount and temperature drive isotopic fractionation.

**Fig. 4:**
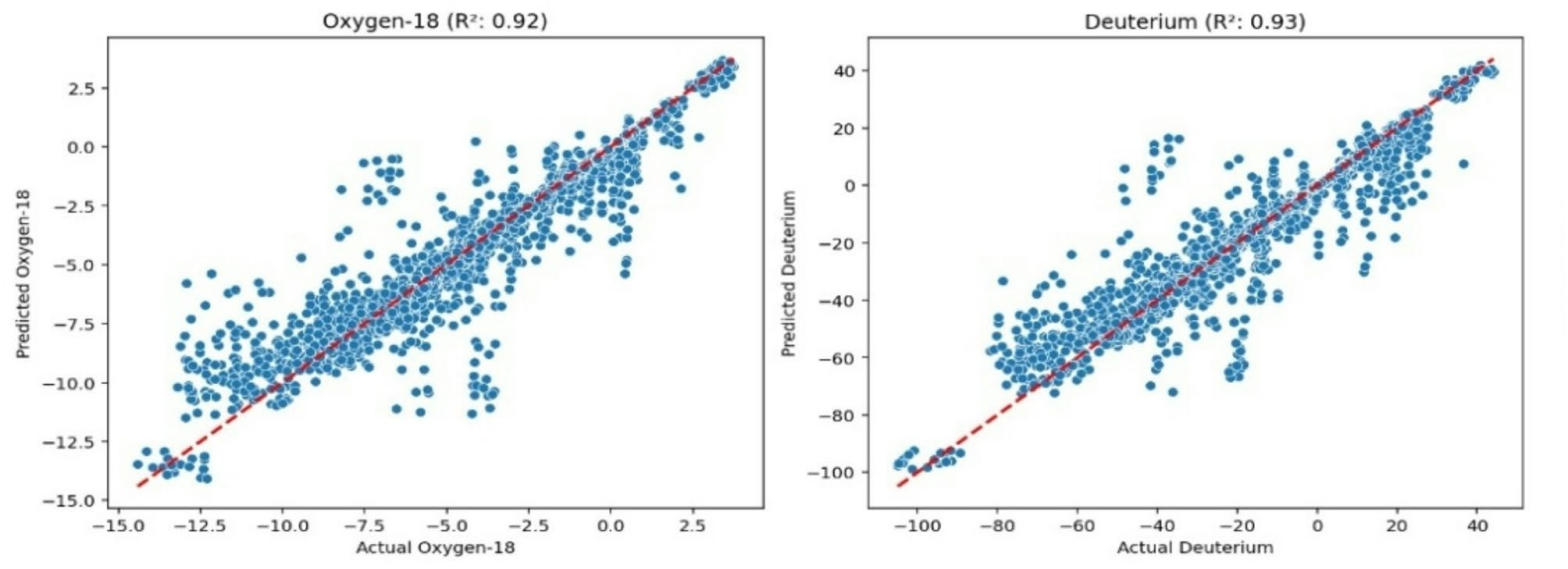
Predicted vs. Actual isotopic values (random forest) data amplification.

To increase the size of the original dataset (276 samples) and avoid overfitting, and to make the trained model more reliable for real-world use, we used data augmentation. Therefore, we created 100 new samples per row and added slight random changes to the input features (including rain amount, temperature, and average RH) by ± 10%, resulting in 27,600 samples. This process expanded the dataset of 279 samples to the 22,300 samples that were used for training the models shown in Fig. 4.

The goal of this process is to expose the machine learning model to a wider variety of data than what was originally available. This helps prevent the model from “memorizing” the training data (a problem known as overfitting) and improves its ability to make accurate predictions on new, unseen data, which includes 5,576 test samples. The high R² values (0.92 for δ^18^O and 0.93 for δ^2^H) in the model charts suggest that this approach was successful, as the models demonstrate a strong predictive capability on the test data.

Figure 4 shows scatter plots comparing the predicted isotopic values against the actual measured values for δ^18^O and δ^2^H, along with their respective R² values. The strong linear correlation (indicated by R² values δ^18^O and δ^2^H) further confirms the high predictive power of the Random Forest model. The data points generally align well with the dashed red line (representing perfect prediction), demonstrating the model’s ability to accurately capture the variability in these isotopic compositions.

## Conclusions and implications

### Superiority of random forest

- RF’s ensemble approach (multiple decision trees) effectively captured non-linear relationships between rainfall variables and isotopes.
- Its low overfitting and high generalizability make it ideal for environmental isotope modeling.

### Key finding for water resources management

- The main outcome is that Random Forest can reliably reconstruct rainfall isotope signatures (δ¹⁸O and δ²H) from routine meteorological data, enabling spatially and temporally continuous isotope information even when direct isotope sampling is limited.

### Practical applications

- The model can be used for hydrological tracing, climate studies, and water resource management in regions with similar rainfall patterns.
- Policymakers can leverage these predictions to assess water origin and movement in ecosystems.
- The model can support groundwater recharge assessment and source identification by providing estimated rainfall isotope inputs for comparison with groundwater and surface-water isotopes.

## Limitations and future work

- Data dependency: Model accuracy relies on high-quality, region-specific rainfall data.
- Algorithm tuning: Further optimization (e.g., hyperparameter tuning) could enhance performance.

For predicting environmental isotopes (δ¹⁸O and δ²H) from rainfall data, Random Forest is the recommended algorithm due to its high accuracy, interpretability, and robustness. Future studies could explore hybrid models (e.g., RF + ANN) or larger datasets to refine predictions.

## Data Availability

“The datasets used and/or analyzed during the current study available from the corresponding author on reasonable request.”
